# The Distribution of the Photoreceptor Outer Segment Length in a Healthy Population

**DOI:** 10.1155/2017/4641902

**Published:** 2017-12-19

**Authors:** Gamze Maden, Akin Cakir, Dilan Icar, Burak Erden, Selim Bolukbasi, Mustafa Elcioglu

**Affiliations:** Eye Clinic, Okmeydani Training and Research Hospital, Istanbul, Turkey

## Abstract

**Purpose:**

To evaluate the effects of age and sex on the photoreceptor outer segment (PROS) length in healthy eyes, using spectral-domain optical coherence tomography (SD-OCT).

**Methods:**

A total of 97 eyes of 97 healthy participants (spherical equivalent < ±1 diopters [D]) were scanned with SD-OCT. The patients were divided into 3 groups by age: group 1 (0–20 years), group 2 (21–40 years), and group 3 (41–60 years). The PROS length was defined as the distance from the inner surface of IS/OS (inner segment/outer segment) band to the inner surface of retinal pigment epithelium (RPE).

**Results:**

The mean PROS length was 52.01 ± 3.79 *μ*m in females and 53.41 ± 3.37 *μ*m in males (*p* = 0.061). The mean PROS length of the different groups was 53.70 ± 3.18 *μ*m (0–20 years), 52.14 ± 3.64 *μ*m (21–40 years), and 52.20 ± 3.95 *μ*m (41–60 years) (*p* = 0.155; ANOVA test). Multiple linear regression analysis revealed a −0.039 *μ*m decline in PROS length per year (*p* = 0.074) and a −1.408 *μ*m decline in females (*p* = 0.055).

**Conclusion:**

The difference in PROS length was not statistically significant neither for age nor for gender; females tended to have a lower PROS length than males, and PROS length was slightly higher in the first two decades of life.

## 1. Introduction

**Figure 1:**
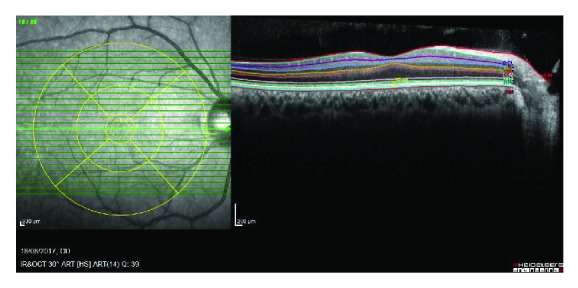
Green-colored line represents IS/OS junction, turquoise-colored line represents RPE, and the distance between these two lines represent PROS length.

The human fovea is the center of photoreceptor topography that lies on the visual axis. It occupies only about 0.02% of the retina; however, it contributes to the visual function the most [1]. This feature makes the fovea distinguished because of the dense cone cells and the absence of the rod cells in this area. These photoreceptors are morphologically specialized cells that have two apical compartments, the inner segment and the outer segment [2]. The outer segment is the part that is filled with light sensitive visual pigment molecules which are constantly renewed. Cone outer segments differ from rods by connecting to the plasma membrane, which also means being open to extracellular space [3]. Thus the outer photoreceptor layer of the fovea (virtually “cone outer segment” may be more accurate) is a very significant trait for vision. Adaptive optics is available for imaging cone density, whereas spectral-domain optical coherence tomography (SD-OCT) is a useful method in imaging cone architecture [4]. There are four hyperreflective outer retinal lines on SD-OCT: external limiting membrane (ELM), inner segment ellipsoid (ISel, previously called the junction between the inner and the outer segments (IS/OS junction)), cone outer segment tips (COST), and retinal pigment epithelium (RPE) [5] (Figure 1). The PROS is located in between IS/OS and RPE.

The PROS length has been mentioned before in the literature. Shiono et al. reported PROS length as a good predictive value for postoperative visual acuity of idiopathic epiretinal membrane patients [6]. Fujihara-Mino et al. revealed the relation between the PROS length and the visual outcomes after anti-VEGF therapy for retinal vein occlusion [7]. Wilk et al. evaluated the outer segment length to estimate the foveal cone density [8]. However, there is an obscurity about the mean PROS length in healthy eyes and how it is altered by age and gender.

To the best of our knowledge, there has been no previous report in the literature related to this issue. Therefore in this study, we intend to evaluate the effects of age and gender on the PROS length in healthy eyes.

## 2. Methods

Ninety-seven eyes of 97 healthy patients were enrolled in this prospective cross-sectional study. This study was approved by the ethics committee of Istanbul University (approval ID: 2017/376) and adhered to the tenets of the Declaration of Helsinki. Written informed consent was obtained from all participants.

We used G^∗^Power 3.1 (Universitat Düsseldorf) statistical power analysis programme to calculate the sample size before conducting the study. We had found out that the study had to recruit 23 individuals for each group to have 95% power with 5% type 1 error level to detect a minimum clinically significant difference of 1 *μ*m (effect size) in PROS length. In accordance with this, healthy patients, who had consulted to our eye clinic and had not met the exclusion criteria listed below, were randomly recruited to the current study. Afterwards, participants were divided into three age groups: group 1 (0–20 years), group 2 (21–40 years), and group 3 (41–60 years).

The patients with any anterior-posterior segment pathology, any refractive errors greater than ±1 D spherical equivalent, the presence of systemic diseases such as diabetes mellitus, hypertension, and connective-tissue diseases, the history of ocular trauma and ocular surgery, and the history of chronic ocular diseases such as glaucoma and uveitis were excluded from the study.

The following examinations were applied to all the participants in the study: visual acuity, anterior segment biomicroscopy, intraocular pressure, fundoscopy, and SD-OCT analysis.

SD-OCT scans were performed with OCT Spectralis (Spectralis, Heidelberg Engineering, Heidelberg, Germany). Merely the right eyes of the subjects were included in the study. The entire macular area was scanned, and the following scan acquisition parameters were required: dense volume scan (30° × 25°, roughly 9 × 7.5 mm), 31 B-scans each spaced 244 ϻm apart, automatic real-time mean of 20, and high speed (512 A-scans/B-scan).

The PROS length was defined as the distance from inner surface of IS/OS band to the inner surface of RPE. After automatic segmentation of all the layers, the measurements were performed manually by using digital calipers at the foveal center (foveola) where the ellipsoid zone and foveal bulging formed (Figure 1). Since, as far as is known, this area was only consisted of the cones. Two experienced observers (GM and DI) who were masked to the patients' information measured the PROS length independently, and the average of the two observers was used for the statistical analyses. We analysed the interobserver and intraobserver correlations. Interobserver variability was evaluated by comparing the two observers' measurements statistically (*p* = 0.161, Student's *t*-test). Intraobserver variability was assessed by looking at the coefficient of variance (% CV). CV was calculated by using the following formula: CV = (SD/mean)∗100. The first observers' CV was 32%, and the second observers' CV was 35%.

Statistical analyses were carried out using SPSS 17.0 software for Windows (SPSS Inc., Chicago, IL). Descriptive analyses were presented using means and standard deviations for normally distributed values. Since the PROS length was normally distributed, Student's *t*-test was used to compare this parameter between the genders. One-way ANOVA was performed to compare this parameter among groups 1, 2, and 3. A multiple linear regression model was used to describe the effects of age and gender on PROS length. *p* value of less than 0.05 was considered to be statistically significant.

## 3. Results

**Figure 2:**
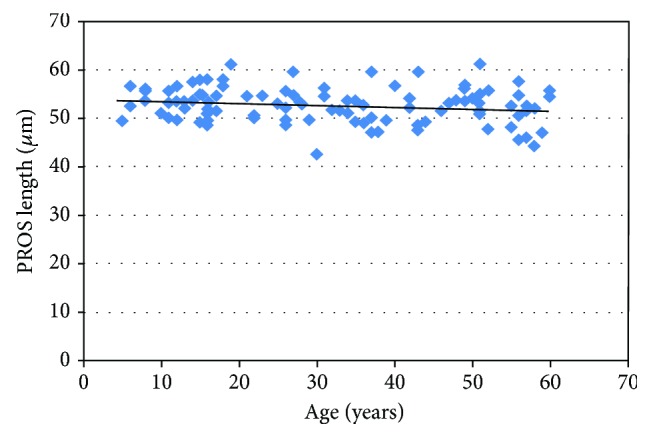
The scatter plot graphic of the photoreceptor outer segment length by age groups.

This study constituted 97 patients with 31 eyes in group 1, 32 eyes in group 2, and 34 eyes in group 3. The demographic characteristics, mean SE, and PROS length values for each group were summarized in Table 1. The mean PROS length was 53.70 ± 3.18 *μ*m in group 1, 52.14 ± 3.64 *μ*m in group 2, and 52.20 ± 3.95 *μ*m in group 3. Although PROS length was found to be slightly higher in group 1, this was not statistically significant (*p* = 0.155) (Figure 2).

**Figure 3:**
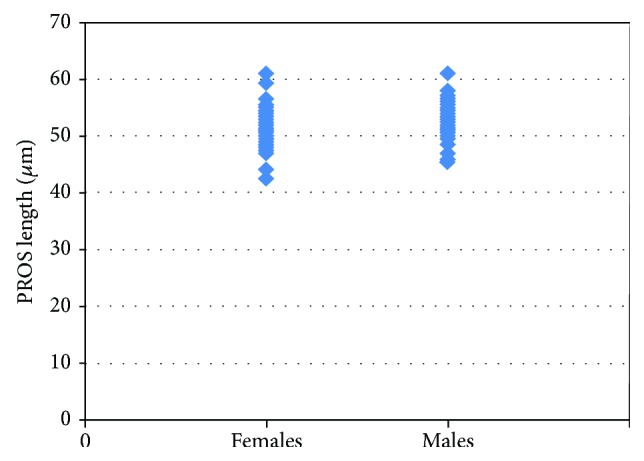
The scatter plot graphic of the photoreceptor outer segment length by gender.

When the patients were assessed totally, 52 of the 97 eyes (53%) turned out to be of female patients. The mean PROS length was 52.01 ± 3.79 *μ*m in females and 53.41 ± 3.37 *μ*m in males. Despite a lower PROS length in females, this was not statistically significant as well (*p* = 0.061) (Figure 3).

Pearson correlation analyses showed a statistically significant but a low strength of association between PROS length and gender and age. (*p* = 0.031, *r* = −0.191; *p* = 0.41, *r* = −0.177; resp.). In the multiple linear regression analysis, a −0.039 *μ*m decline in PROS length per year and a −1.408 *μ*m decline in females were remarkable. However, those did not reach statistical significance as well (*p* = 0.074 and *p* = 0.055, resp.).

## 4. Discussion

Recent advances in ocular imaging technology have successfully introduced novel analyses to retina morphology. Our study investigates the average PROS length in different age and gender groups within the otherwise healthy population. In the present study, even if it did not acquire a statistical significance, we have observed that age and gender have a partial effect on the PROS length. We found that women's PROS length was lower than men's and the PROS length of the participants between the ages of 0–20 years was mildly higher than the others. Additionally, PROS length was slightly reduced per year.

To the best of our knowledge, there is no previous epidemiologic report in the literature based on the effects of age and gender on the PROS length. Some normative data studies had been published, but those had conflicting results. Also those did not focus on the PROS length as well as ours but rather focused on the outer retinal layer thickness. In our study, the mean PROS length was 52.01 ± 3.79 *μ*m in females and 53.41 ± 3.37 *μ*m in males. In addition, the mean PROS length of the different groups was 53.70 ± 3.18 *μ*m (0–20 years), 52.14 ± 3.64 *μ*m (21–40 years), and 52.20 ± 3.95 *μ*m (41–60 years). Shin et al. found out that the mean outer photoreceptor layer (OPRL) thickness in normal eyes in all subfields was 40.37 ± 4.35 *μ*m [5]. Christensen et al. reported that the mean OPRL thickness of the foveal center was 77.2 *μ*m [9]. Bagci et al. documented that the mean OPRL thickness was 35 ± 4 *μ*m, as determined by their own segmentation algorithm on SD-OCT [10]. The main reason for the difference in the results was the fact that the determination of the OPRL varied across studies. Another reason was that those studies consisted of limited and older subjects, in short forementioned researches did not take the participants' age and gender into consideration.

We want to underline the fact that we measured the length of the cone outer segment (COS) rather than PROS because we made our measurements exactly at the fovea which consists of only the cone outer segments. Nevertheless, we used the term “PROS” similar to the previous studies in the literature, but a new terminology like “COS” may be more accurate. There is a similar report in the literature. Wilk et al. reported that cone density was significantly correlated with PROS length in patients with albinism [8]. Additively, they observed no significant association between PROS length and cone density of normal subjects. They explained this result as the following: the most likely reason for this is that some of the normal subjects' peak cone densities will be underestimated due to the inability to identify every cone in the mosaics of the highest density. This is supported by the fact that in vivo adaptive optics- (AO-) derived estimates of peak cone density, on average, fall below those reported from histology [11].

There were also some publications that studied the PROS as a predictor factor in different retinal pathologies. Hashimoto et al. reported that BCVA was significantly correlated exclusively with the elongated foveal PROS in macular hole surgery [12]. In another study, Shiono et al. declared that the PROS length was a good predictor of postoperative visual acuity after surgery for idiopathic epiretinal membrane [6]. Also, Fujihara-Mino et al. found out that the PROS length after the macular edema resolution was significantly correlated with the BCVA and the retinal sensitivity at the final visit [7]. We agree with the aforementioned authors that the PROS length may be a predictor factor for not only visual acuity but also contrast sensitivity, color vision, or other signs of visual perception. Therefore, knowing the normal values of PROS may contribute to the literature in regard to the prognosis of different retinal diseases.

Since we calculated the sample size prior to the study, we think that our study population was adequate to interpret our findings. Also, we excluded the patients with any refractive errors greater than ±1 D spherical equivalent in order to minimize the effect of axial length on our results. However, the main basic limitation of the current study is that the measurements of PROS were performed manually. The superior and inferior borders (IS/OS layer and RPE, resp.) were drawn automatically by the programme (segmentation of all layers) embedded in Spectralis OCT, and we acquired the average values obtained by two experienced observers and considered the interintra observer variability. Still a bias may have influenced our results. Surely, it would be more accurate if it was possible to measure PROS length automatically by the programmes embedded in the SD-OCT instrument such as the values retinal nerve fiber layer, ganglion cell layer, and internal plexiform layer. A full automated image analysis program for PROS length measurement would provide more accurate data.

Further comparative studies on the PROS length in specific disease groups will be more useful for literature. We believe that our study will inspire many new researchers about PROS or COS.

## Figures and Tables

**Table 1:** The demographic characteristics, the mean spherical equivalent, and the photoreceptor outer segment length values for each group are shown.

	Group 1 (0–20 years) *n* = 31	Group 2 (21–40 years) *n* = 32	Group 3 (41–60 years) *n* = 34
Age (years; ±SD)	13.03 ± 3.85	30.50 ± 5.52	51.58 ± 5.56
Gender (female %)	51%	56%	52%
Spherical equivalent (mean)	−0.005 ± 0.60	−0.14 ± 0.47	0.27 ± 0.42
Photoreceptor outer segment (PROS) length (mean; *μ*m)	53.70 ± 3.18	52.14 ± 3.64	52.20 ± 3.95
